# Visualization of self-delivering hydrophobically modified siRNA cellular internalization

**DOI:** 10.1093/nar/gkw1005

**Published:** 2016-11-29

**Authors:** Socheata Ly, Deanna M. Navaroli, Marie-Cécile Didiot, James Cardia, Lakshmipathi Pandarinathan, Julia F. Alterman, Kevin Fogarty, Clive Standley, Lawrence M. Lifshitz, Karl D. Bellve, Matthieu Prot, Dimas Echeverria, Silvia Corvera, Anastasia Khvorova

**Affiliations:** 1 Program in Molecular Medicine, University of Massachusetts Medical School, Worcester, MA 01655, USA; 2 RNA Therapeutics Institute, University of Massachusetts Medical School, Worcester, MA 01655, USA; 3 RXi Pharmaceuticals, Marlborough, MA 01752, USA

## Abstract

siRNAs are a new class of therapeutic modalities with promising clinical efficacy that requires modification or formulation for delivery to the tissue and cell of interest. Conjugation of siRNAs to lipophilic groups supports efficient cellular uptake by a mechanism that is not well characterized. Here we study the mechanism of internalization of asymmetric, chemically stabilized, cholesterol-modified siRNAs (sd-rxRNAs^®^) that efficiently enter cells and tissues without the need for formulation. We demonstrate that uptake is rapid with significant membrane association within minutes of exposure followed by the formation of vesicular structures and internalization. Furthermore, sd-rxRNAs are internalized by a specific class of early endosomes and show preferential association with epidermal growth factor (EGF) but not transferrin (Tf) trafficking pathways as shown by live cell TIRF and structured illumination microscopy (SIM). In fixed cells, we observe ∼25% of sd-rxRNA co-localizing with EGF and <5% with Tf, which is indicative of selective endosomal sorting. Likewise, preferential sd-rxRNA co-localization was demonstrated with EEA1 but not RBSN-containing endosomes, consistent with preferential EGF-like trafficking through EEA1-containing endosomes. sd-rxRNA cellular uptake is a two-step process, with rapid membrane association followed by internalization through a selective, saturable subset of the endocytic process. However, the mechanistic role of EEA1 is not yet known. This method of visualization can be used to better understand the kinetics and mechanisms of hydrophobic siRNA cellular uptake and will assist in further optimization of these types of compounds for therapeutic intervention.

## INTRODUCTION

A broad range of human diseases, including cancer, infection and neurodegeneration, can be treated via the silencing of specific genes using small oligonucleotides. Oligonucleotide therapeutics (ONTs) are a new class of drugs that are distinguished by targeting DNA or RNA directly, thus preventing expression of the protein responsible for the disease phenotype (1–3). Advantages of ONTs over conventional drugs include ease of drug design based solely on base-pairing rules, the ability to access targets previously considered ‘undruggable’, and their promise of unprecedented specificity, potency, and duration of effect (4). In addition, the pharmacokinetics, pharmacodynamics, and safety of ONTs are mostly defined by chemical modifications/formulation (5); these characteristics tend to be very similar between compounds targeting different genes, enabling multi-gene silencing and simple development of drugs targeting specific tissues (6). Significant effort in the last decade resulted in the development of several types of both chemically-modified and formulated ONTs with clear clinical efficacy (7). Thus, ONTs represent a new and potentially transformative therapeutic paradigm. Nonetheless, their clinical utility has been mostly limited to hepatocyte delivery (8) and local administration (9).

siRNAs comprise one of the major classes of ONTs. These small double-stranded oligonucleotides consist of guide (antisense) and passenger (sense) strands and utilize the RNA interference (RNAi) pathway (10). Upon cellular uptake, the guide strand is loaded into an RNA induced silencing complex (RISC) capable of cleaving its complementary target RNA. The number of loaded RISCs per cell sufficient to induce efficient and long-term gene silencing is estimated at ∼25-100 *in vitro* (11) and ∼400 *in vivo* (12). Usually 10–100 ng siRNA/g of tissue is enough to generate sufficient active RISCs to induce silencing (12). Loaded RISCs have week-long stability, resulting in prolonged gene silencing (3-6 weeks) from a single administration (7).

Oligonucleotides are charged non-biologically stable molecules which need to be modified or formulated to enable cellular delivery. Furthermore, their efficacy is defined by both the ability to be delivered to cells and tissues as well as biological availability inside the cell, with the vast majority of internalized compounds being trapped unproductively in lysosomes and other ‘oligonucleotide sinks’ (13–15). Conjugation of stabilized siRNAs to bioactive conjugates has been shown to promote activity both *in vivo* and *in vitro*. Despite years of exploration, only a limited number of formulations and conjugates have progressed to the clinic: lipid nanoparticles (LNPs) (16,17), dynamic polyconjugates (18), dextrins (19), N-acetylgalactosamine (GalNAc) (8) and cholesterol (9). The most clinically advanced delivery platform is GalNAc-conjugated siRNA (GalNAc-siRNA) with several compounds showing robust clinically efficacy (20–22). Trivalent GalNAc ligands facilitate the uptake of GalNAc-siRNAs into hepatocytes through an interaction with asialoglycoprotein receptors (ASGPRs) overexpressed on the surface of hepatocytes. While promising, both GalNAc-siRNAs and LNPs are mostly limited to liver-associated delivery and diseases. Alternatively, lipophilic conjugates promote efficient cellular internalization independently of cell type, with one compound being currently evaluated for the treatment of hypertrophic scarring following local skin administration (23). While the mechanism of GalNAc-siRNAs (24) and LNP (13,25–27) formulated siRNA uptake is relatively well characterized, only limited data exists regarding the internalization of lipophilic siRNAs (28,29). Wolfrum *et al*. (30) have demonstrated that a fraction of cholesterol modified siRNAs binds to different lipoproteins in blood and LDL/HDL complexes associate with cells and tissues, which has also been repeated with other hydrophobically modified oligonucleotides (31,32). It is unclear if the mechanism of cellular intrnalization *in vivo* and *in vitro* is similar since *in vitro*, serum and LDL/HDL strongly inhibit functional efficacy of cholesterol modified siRNAs, with the best efficacy observed in serum-free media (Supplementary Figure S1). Here, we decided to systematically visualize the internalization of the lipophilic siRNAs using small, asymmetric, hydrophobically modified siRNAs called sd-rxRNAs (9,33).

We show that sd-rxRNAs are quickly internalized through the formation of vesicular structures, indicative of endocytosis. Given that inefficient delivery of siRNA to the site of action inside the cell remains one of the major limitations of RNAi-based drugs, dissecting the precise intracellular trafficking mechanisms of conjugated siRNAs could elucidate the functional relevance of these pathways. Furthermore, recent results determined that early endosomes are heterogeneous, displaying different sizes, motilities, and phosphoinositide effector complements (34–36). These endosomes may be specialized for the trafficking of different cargos, as receptors for transferrin (Tf) and epidermal growth factor (EGF) are found in different subsets of endosomes almost immediately after internalization (37). Moreover, the genetic networks involved in the early trafficking of the Tf and EGF receptors differ substantially from each other (38). Directly distinguishing specific internalization pathways requires high temporal and spatial resolution to visualize the movement of ligands from the outside to the inside of the cell, and amongst distinct early endocytic structures. We used a recently developed microscopy platform that combines total internal reflection fluorescence (TIRF) and structured illumination microscopy (SIM) to detect the position of endosomes relative to the plasma membrane during the uptake of ligands (39). Using this methodology, we find that sd-rxRNA oligonucleotides internalize selectively through a pathway similar to that taken by the EGF receptor, which utilizes endosomes enriched in the phosphoinositide-binding protein EEA1 (37,40–43). In addition, we demonstrate that the second step of intracellular localization is saturable, further supporting the notion of potential chemistry-guided endosome sorting. Determination of the mechanism of oligonucleotide uptake is important for enhancing both *in vitro* and *in vivo* efficacy. The recognition that hydrophobic oligonucleotide uptake involves distinct intracellular endosomal pathways and is saturable will aid in the development of rational strategies to enhance the potency and utility of RNAi-based therapeutics.

## MATERIALS AND METHODS

### Reagents

Polyclonal EEA1 antibody was produced in chickens by injecting N-terminal 6-HIS fusion protein of human EEA1 residues 32–218. Polyclonal RBSN antibody was produced in rabbits by injecting residues 137–784 of human RBSN. Unconjugated and DyLight-649 conjugated human transferrin were obtained from Jackson Immunochemicals. FM4-64 (T3166), Alexa Fluor-labeled epidermal growth factor (E13345, E35351) and Alexa Fluor-labeled transferrin (T13342, T13342) were obtained from Life Technologies.

### Cell culture and transfection

COS-7 cells were maintained in DMEM (Invitrogen) supplemented with 100 U/ml penicillin streptomycin (Invitrogen), 0.1 mg/ml normocin (InvivoGen) and 10% fetal bovine serum (Atlanta Biologicals) at 37°C and 5% CO_2_. Cells were seeded at a density of 1 × 10^5^ cells per dish in 35 mm dishes (MatTek P35G-0.170-14-C) and grown for 24 h. For transfections, calcium phosphate was used to transfect 1 μg DNA. The cells were then washed with fresh media after 16 hours and allowed to grow for an additional 24 h. Live cell imaging was done in FluoroBrite DMEM (Thermo Fisher A1896702) within an environmental chamber to maintain 37°C, 5% CO_2_ and 100% humidity.

### TIRF/epifluorescence structured illumination microscope (TESM) optical system

A custom-built microscope system, TESM, simultaneously combines total internal reflection fluorescence (TIRF) and wide-field epifluorescence modes and incorporates structured illumination microscopy (SIM) in the epi mode for fast optical sectioning and enhanced spatial resolution. Further details as well as the TESM acquisition system is described previously (39). Image stacks acquired with SIM were 10 μm tall with slices spaced 100 nm apart. For co-localization analysis, dual-labeled image sets were convolved with a difference of Gaussians (DOG) filter consisting of (i) a small, two dimensional, Gaussian spot with unit area (sigma = 150 nm) that acted as a vesicle matched detector, i.e. an approximation to a near-diffraction limited spot and (ii) a larger, inverted, two dimensional Gaussian (sigma = 300 nm) with negative unit area that estimated and subtracted the local background. The Gaussian smoothed images were visually thresholded (global threshold) to select for pixels belonging to objects (e.g. vesicles) and eliminate areas devoid of signal (but containing noise). For each label, its globally thresholded image was used to generate a binary image by setting the intensity of all positive value pixels to one and all other pixels to zero. Co-localization was determined by two-way or three-way binary image overlap among the labels. Co-localization values are reported as the percent of pixels that are co-localized with a given label: given a pair of 2D binary images of labels A and B, the percent co-localization of ‘B with A’ at time *t* is:

\begin{equation*}100 \times \sum {\sum {[\mathop {{\rm{A}}({{x}},{{y}},{{t}})}\limits_{{{x}}\;{{y}}} \times {\rm{B}}({{x}},{{y}},{{t}})]} {\rm{/}}\sum {\sum {\mathop {{\rm{B}}({{x}},{{y}},{{t}})}\limits_{{{x}}\;{\rm{y}}} } } } \end{equation*}

Additionally, non-specific co-localization was calculated by rotating one of the channels by 180° and re-calculating the co-localization using the same equation. This allowed us to determine that co-localization was genuine and not simply due to excess signal.

### Confocal microscopy

Confocal images of sd-rxRNA uptake in HeLa cells were acquired with a Leica DM IRE2 confocal microscope using a 63× oil immersion objective. Images were processed in ImageJ (v1.50d). For sd-rxRNA uptake, total fluorescence intensity was measured. For measuring sd-rxRNA in the perinuclear region, a line was drawn through the longest part of the cell that intersects the nucleus. sd-rxRNA fluorescence intensity was measured on this line 10 pixels from the nucleus on both sides. Likewise, plot profiles were also acquired in ImageJ using the same lines.

### DeltaVision OMX v4 blaze microscope

Super-resolution 3D-SIM images were acquired on a DeltaVision OMX V4 (GE Healthcare) equipped with a 60×/1.42 NA PlanApo oil immersion lens (Olympus), 405, 488, 568 and 642 nm solid state lasers and sCMOS cameras (pco.edge). Image stacks of 10 μm with 0.125 μm thick z-sections and 15 images per optical slice (three angles and five phases) were acquired using immersion oil with a refractive index of 1.514. Images were reconstructed using Wiener filter settings of 0.008 for the 405 channel, 0.006 for the 528 channel, and 0.003 for the 508 and 683 channels. Optical transfer functions (OTFs) were measured specifically for each channel with SoftWoRx 6.1.3 (GE Healthcare) to obtain super-resolution images with a 2-fold increase in resolution both axially and laterally. Images from different color channels were registered using parameters generated from a gold grid registration slide (GE Healthcare) and SoftWoRx 6.5.2 (GE Healthcare). Volume renderings were generated in ImageJ v1.50g.

### Gene expression analysis

The QuantiGene 2.0 bDNA assay (Affymetrix, QS0011) was used to perform the PPIB sd-rxRNA dose response. HeLa cells, resuspended in DMEM with 6% FBS without antibiotics, were added into each well of a 96-well plate with a density of 5000 cells/50 μl per well. sd-rxRNA was diluted to 2× final concentration in serum free OptiMEM and 50 μl of compound was added to each well for a final volume of 100 μl/well and a final concentration of 3% FBS. The mixture was incubated under standard conditions for 72 h and then cells were lysed and processed according to the manufacturer's recommended protocol. Briefly, cells were lysed in 250 μl diluted lysis mixture (1:2 lysis mixture:water) with 0.167 ug/μl proteinase K (Affymetrix QS0103) for 30 min at 55°C. Cell lysates were mixed thoroughly and 20–80 μl of lysate was added to a bDNA capture plate along with 0–60 additional diluted lysis mixture without proteinase K to fill up to 80 μl total volume. Probe sets were diluted as specified in Affymetrix protocol and 20 μl of either human HPRT, PPIB, EEA1 or RBSN probes (Affymetrix: SA-50339, SA-10003, SA-13403, SA-21039, respectively) were added to each well of capture plate to a final volume of 100 μl. Luminescence was detected on a Tecan M 1000.

### Oligonucleotide synthesis

Supplementary Table S1 lists the species used in this study. Therapure RNA phosphoramidite monomers were procured from Thermo Fisher Scientific. Synthesis solid supports (CPG) attached to the first 3′ residue were obtained from Prime Synthesis (Aston, PA). Synthechol (cholesterol) was subjected to multi step synthesis to yield Chol-TEG-Gly succinate (work was performed at Organix, Woburn, MA, USA) and loaded onto CPG (conducted by Prime Synthesis Aston PA). Phosphitylation reagent for 5′ phosphorylation was procured from ChemGenes (Wilmington, MA, USA). All other synthesis reagents and solvents were obtained from American International Chemicals and used as such. Other chemicals and solvents for post synthesis workflow were purchased from Sigma Aldrich and used without any purification or treatment. Oligonucleotides were synthesized by RXi Pharmaceuticals or UMass using standard synthesis protocols.

RNA oligonucleotides were synthesized on a Mermade 12 DNA/RNA Synthesizer (Bioautomation, Plano, Texas) using standard oligonucleotide phosphoramidite chemistry starting from the 3′ residue of the oligonucleotide preloaded on CPG support. Synthesized oligonucleotides were cleaved from the support and the protecting groups were removed by the successive treatments of 3:1 aqueous ammonia:ethanol (65°C, 2 h) (Sigma, St. Louis, MO, USA) and triethylamine-trihydrofluoride (DMSO, 70°C, 40 min) (Sigma, St. Louis, MO). Crude Oligonucleotides were precipitated with isopropanol (Fisher Scientific, Pittsburgh, PA, USA) and centrifuged to obtain a pellet. Crude product was then purified on GE Akta Purifier UPC100 using ion exchange chromatography (Source 15Q, 20 mM NaH_2_PO_4_ (Fisher Scientific, Pittsburgh, PA), 15% CH_3_CN (Merck, Billerica, MA, USA), 1 M NaBr (Sigma, St. Louis, MO), gradient 20–60% B over 30 column volumes) and fractions were analyzed by reverse phase ion pair chromatography on a Shimadzu HPLC. Pure fractions were pooled and desalted by Tangental Flow Filtration using Sius PES 3000 MWCO (High Purity New England, Providence, RI, USA) and evaporated to dryness on GeneVac Personal Evaporator (SP Industries, Warminster, PA, USA). The purity and molecular weight were determined by HPLC analysis (Shimadzu Prominence, XBridge OST C18 column, 25 mM hexylammonium acetate-acetonitrile (Sigma, St. Louis, MO) buffer system, 60°C) and ESI-MS analysis using Promass Deconvolution for Xcalibur (Novatia, Monmouth Junction, NJ, USA).

## RESULTS

### sd-rxRNA quickly internalizes through endocytic pathways following membrane association

**Figure 1.**
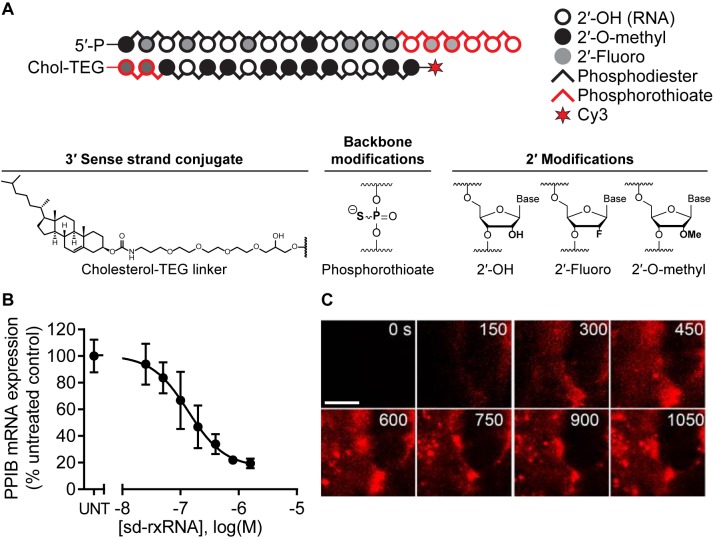
Structure of sd-rxRNA, gene silencing efficacy, and cellular internalization. (**A**) Chemical modifications of sd-rxRNA include 2′-*O*-methyl, 2′-fluoro, phosphorothioate linkages, and cholesterol covalently linked at the 3′ end of the sense strand by a tetraethylene glycol (TEG) linker. (**B**) Dose response was performed in HeLa cells using *PPIB*-targeting sd-rxRNA (UNT: untreated cells). mRNA levels were measured by QuantiGene Assay (Affymetrix). (**C**) COS-7 cells were treated with 250 nM sd-rxRNA-DY547 (red) and visualized by total internal reflection (TIRF) microscopy (see Supplementary Video 1). Scale bar = 10 μm.

sd-rxRNAs are asymmetric compounds with a short duplex region (15 base pairs) and a single-stranded tail which is typically phosphorothioated (PS). In addition, chemical modifications are incorporated throughout the compound, including 2′-fluoro and 2′-*O*-methyl modifications (providing stabilization and avoidance of PKR response), and the 3′ end of the passenger strand may be conjugated to TEG-Cholesterol (Figure 1A). The cholesterol enables quick membrane association while the single-stranded PS tail is essential for cellular internalization by a mechanism similar to that used by conventional antisense oligonucleotides (30,44–45). Both the cholesterol and PS tail are essential for *in vitro* activity; without the cholesterol conjugate, the oligonucleotide shows very little activity and uptake in HeLa cells (Supplementary Figure S2). Addition of Cy3-labeled sd-rxRNA to any cultured cell type shows quick and efficient internalization (data for COS-7 cells shown in Figure 1C) and gene silencing (Figure 1B). Compounds are effective *in vivo* upon local administration in skin (23), eye (9), and brain (46). The compound targeting CTGF is now in Phase II clinical trials to prevent or reduce hypertrophic scar formation following scar revision surgery (47, https://clinicaltrials.gov/ct2/show/NCT02030275). Internalization *in vitro* is independent of extracellular factors, such as LDL and other serum components which shift dose response curves ∼3-5 fold (data not present), suggesting that sd-rxRNA contains the chemical features required for cellular association and internalization. Interestingly, lipoproteins have been implicated in cholesterol modified delivery *in vivo* (30–32), indicating that *in vivo* and *in vitro* mechanisms of uptake might be different.

Here, we first visualize the uptake of sd-rxRNA into COS-7 cells using TESM. Fluorescently-labeled sd-rxRNA targeting MAP4K4 (sd-rxRNA-DY547) (9) was added to COS-7 cells at 250 nM and uptake was visualized using live cell TIRF microscopy (see Methods, Figure 1C, Supplementary Video 1). Initial diffuse membrane association is observed within 15 seconds of oligonucleotide exposure followed by the formation of clear, vesicular structures. Within 15 min, a major fraction of fluorescent sd-rxRNA is internalized with vesicles showing semi-random movement in the perinuclear space with the nucleus being clearly visible as oligonucleotide-free (Supplementary Video 1). Similar uptake can be observed in the presence and absence of serum with serum actually inhibiting uptake (Supplementary Figure S1), indicating that initial membrane association is primarily driven by cholesterol hydrophobicity and direct integration into the membrane or interaction with membrane components rather than binding to serum proteins followed by internalization. These data are consistent with previous studies demonstrating that the presence of serum is inhibitory to hydrophobically modified siRNA efficacy *in vitro* (28,29).

Due to their cholesterol moiety, sd-rxRNAs are highly hydrophobic and efficiently associate with plasma membranes; thus, its subsequent internalization might be mediated by bulk phase pinocytosis. The lipophilic styryl dye, *N*-(3-triethylammoniumpropyl)-4-(*p*-diethylaminophenyl-hexatrienyl)pyridinium dibromide (FM4-64), has been extensively used as a vital stain to follow bulk membrane internalization as well as the formation of endocytic vesicles (48–53). It is inserted into the outer leaflet of the plasma membrane, where its interactions with lipids result in strong fluorescence in the far-red spectrum. COS-7 cells were simultaneously treated with 250 nM sd-rxRNA-DY547 and 0.5 μg/ml FM4-64 and visualized using live cell TIRF microscopy. Within seconds of addition to the medium, both sd-rxRNA-DY547 and FM4-64 were both seen labeling the cell in a diffuse pattern, consistent with their partitioning into the hydrophobic plasma membrane. While there is some co-localization, sd-rxRNA also formed puncta distinct from FM4-64 (Supplementary Figure S3, Supplementary Video 2). These results suggested that another pathway might be predominantly contributing to sd-rxRNA trafficking.

### sd-rxRNA cellular internalization, but not membrane binding, is saturable

**Figure 2.**
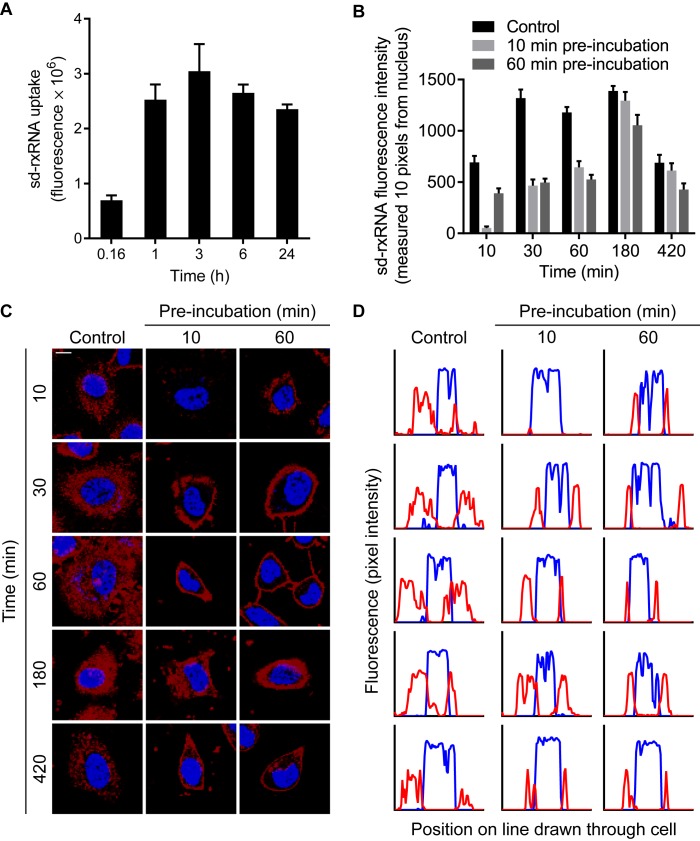
sd-rxRNA is internalized through a saturable pathway. (**A**) HeLa cells were treated with 0.5 μM sd-rxRNA-Cy3 and imaged by live confocal microscopy. Uptake was quantified by measuring total fluorescence intensity at specific time points. (**B**) HeLa cells were either untreated (control) or pre-incubated with 2 μM unlabeled sd-rxRNA for 10 or 60 min. Cells were then treated with 0.5 μM sd-rxRNA-Cy3 and imaged by live cell confocal microscopy. sd-rxRNA fluorescence intensity was measured in a region only 10 pixels from the nucleus. (**C**) Representative images used for quantification in panels **B** and **D**. Blue represents DAPI stain and red represents Cy3-labeled sd-rxRNA. Scale bar = 10 μm. (**D**) Corresponding plot profiles of lines drawn through images in panel **C**. Blue lines represent DAPI stain intensity and red lines represent Cy3-labeled sd-rxRNA intensity.

When sd-rxRNA is added to cellular media, uptake is almost immediate; by 24 h, a small fraction of compounds present in the media is associated with the cells and continuous exposure does not significantly increase the amount of internalized compound (Figure 2A). In order to determine whether this endocytic process is saturable, we exposed HeLa cells to 2 μM of unlabeled sd-rxRNA for either 10 or 60 min, chased by the addition of 0.5 μM fluorescently labeled oligonucleotide. The kinetics of sd-rxRNA-Cy3 uptake in naïve and pre-treated cells was imaged by live cell confocal microscopy (Figure 2C). In naive cells, consistent with data presented in Figure 1 (Supplementary Video 1), sd-rxRNA-Cy3 internalization could be easily detected within 10 min, with oligonucleotide bound to the membrane and distributed in the cytoplasm. Conversely, pre-incubation with unlabeled sd-rxRNA effectively blocks sd-rxRNA-Cy3 cellular internalization (Figure 2B). By 60 min, both control and pre-treated cells showed a dramatic increase in uptake, but the control cells were filled with sd-rxRNA whereas in the pre-treated cells, the majority of sd-rxRNA localized to the membrane and not in the perinuclear region (Figure 2C and D). This effect persisted for several hours; however, by 180 min, sd-rxRNA uptake in both conditions had settled to similar levels, indicating that by 3 h, the factor limiting internalization is recycled back or resynthesized. Taken together, these results suggest that the cellular uptake sd-rxRNA is a two-step process: first, association with the plasma membrane by a lipophilic and/or protein interaction and second, internalization by a saturable endocytic mechanism.

### sd-rxRNA preferentially traffics with EGF compared to Tf by live cell TIRF imaging

In order to further investigate the trafficking mechanism of sd-rxRNA, we compared the cellular uptake with respect to the classical trafficking markers EGF and Tf, whose trafficking pathways are well-defined and, in general, do not overlap. EGF goes to late endosomes/lysosomes for degradation, whereas Tf is recycled back to the plasma membrane (37,38). To this end, we used sd-rxRNA-Cy3 in conjunction with Alexa Fluor 647-labeled EGF (EGF-Alexa647) and Alexa Fluor 488-labeled Tf (Tf-Alexa488). COS-7 cells were simultaneously treated with 250 nM sd-rxRNA-Cy3, 2 ng/μl EGF-Alexa647 and 25 ng/μl Tf-Alexa488. After 10 min, the cells were washed with fresh media to remove unbound ligand. Uptake was visualized continuously by TIRF microscopy (Supplementary Video 3).

**Figure 3.**
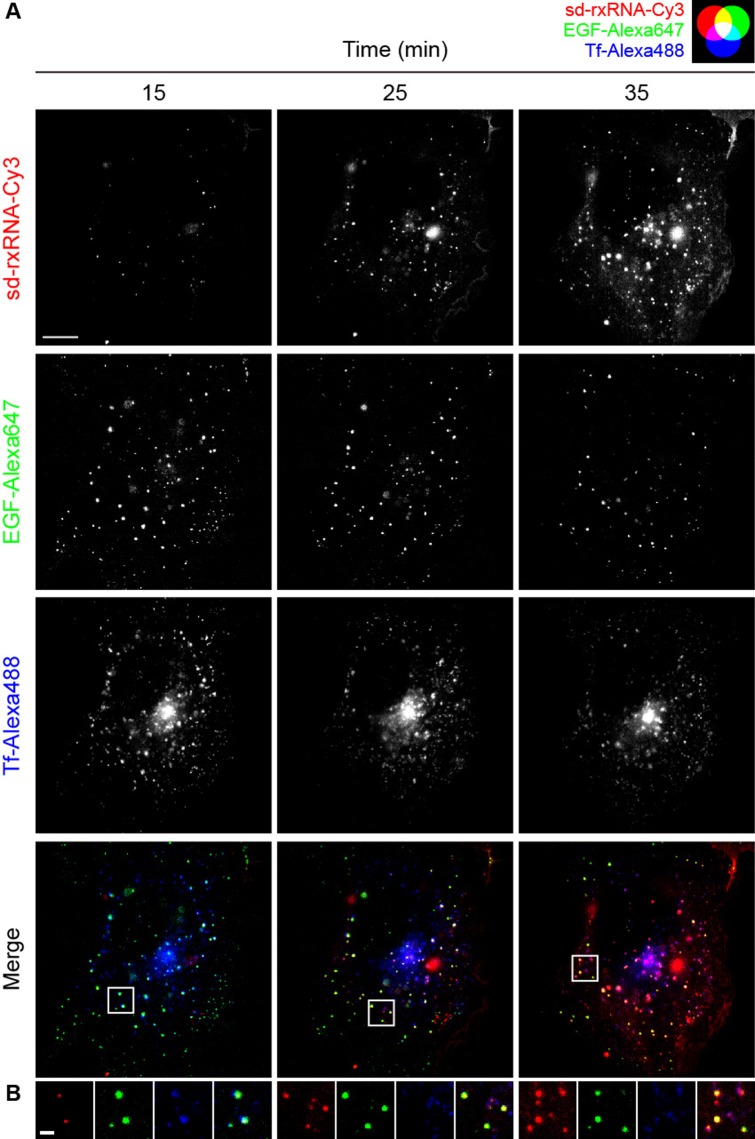
sd-rxRNA preferentially associates with EGF compared to Tf by live cell TIRF. (**A**) COS-7 cells were simultaneously treated with 250 nM sd-rxRNA-Cy3, 2 ng/μl EGF-Alexa647, and 25 ng/μl Tf-Alexa488. After 10 min, the cells were washed with fresh media and imaged by TIRF. Representative time-lapse images are shown (see Supplementary Video 3). Scale bar = 10 μm. (**B**) Enlarged images from panel **A**. Scale bar = 2 μm.

As previously reported, EGF-Alexa647 bound to discrete regions of the plasma membrane distributed at the cell periphery (54) while sd-rxRNA displayed a homogenous distribution on the plasma membrane which intensified over time (Figure 3A). Over the course of the experiment, sd-rxRNA-Cy3 and EGF-Alexa647 foci were clearly co-localizing and moving together, likely inside endosomes (Figure 3B). These results indicate that sd-rxRNA internalizes, at least in part, through a trafficking pathway also involved in EGF uptake.

Conversely, Tf-Alexa488 rapidly bound to the plasma membrane onto variably sized punctate regions due to the concentration of receptors in clathrin-coated pits and patches on the plasma membrane (Figure 3A) (55,56). Tf was subsequently internalized into highly dynamic endocytic structures of different shapes and sizes and accumulated in a compact region near the nucleus known as the recycling compartment. In contrast, sd-rxRNA-Cy3 homogenously labeled the plasma membrane and gradually concentrated over time. This behavior is consistent with a non-specific partitioning of sd-rxRNA-Cy3 into the plasma membrane, likely attributable to the hydrophobic cholesterol moiety. sd-rxRNA-Cy3 and Tf-Alexa488 could be seen in distinct vesicular structures, suggesting that the level of overlap in trafficking pathways is minimal (Figure 3B).

### Quantitative analysis of sd-rxRNA co-trafficking with Tf and EGF shows preferential co-localization with EGF by fixed cell structured illumination microscopy

While live cell imaging shows clear differences between the degree of sd-rxRNA co-localization with EGF and Tf, we next moved to confirm these results using more quantitative methods, allowing evaluation of plurality of cells simultaneously, rather than one cell at a time. COS-7 cells were simultaneously treated with sd-rxRNA-Cy3, 1 ng/μl EGF-Alexa488 and 25 ng/μl Tf-Alexa647 and washed with fresh media at 5 min, allowing simultaneous visualization of all three ligands. At different time points, cells were fixed and optical sectioning images were acquired by SIM. Co-localization analysis was performed on 500 nm optical slices and integrated for the whole cell. Non-specific co-localization was estimated by rotating one channel by 180 degrees and was always below 1% (see Methods for details), confirming that observed co-localization is indeed biologically relevant. For each time point, 10–20 cells were analyzed and a two-tailed Student's *t*-test was performed.

**Figure 4.**
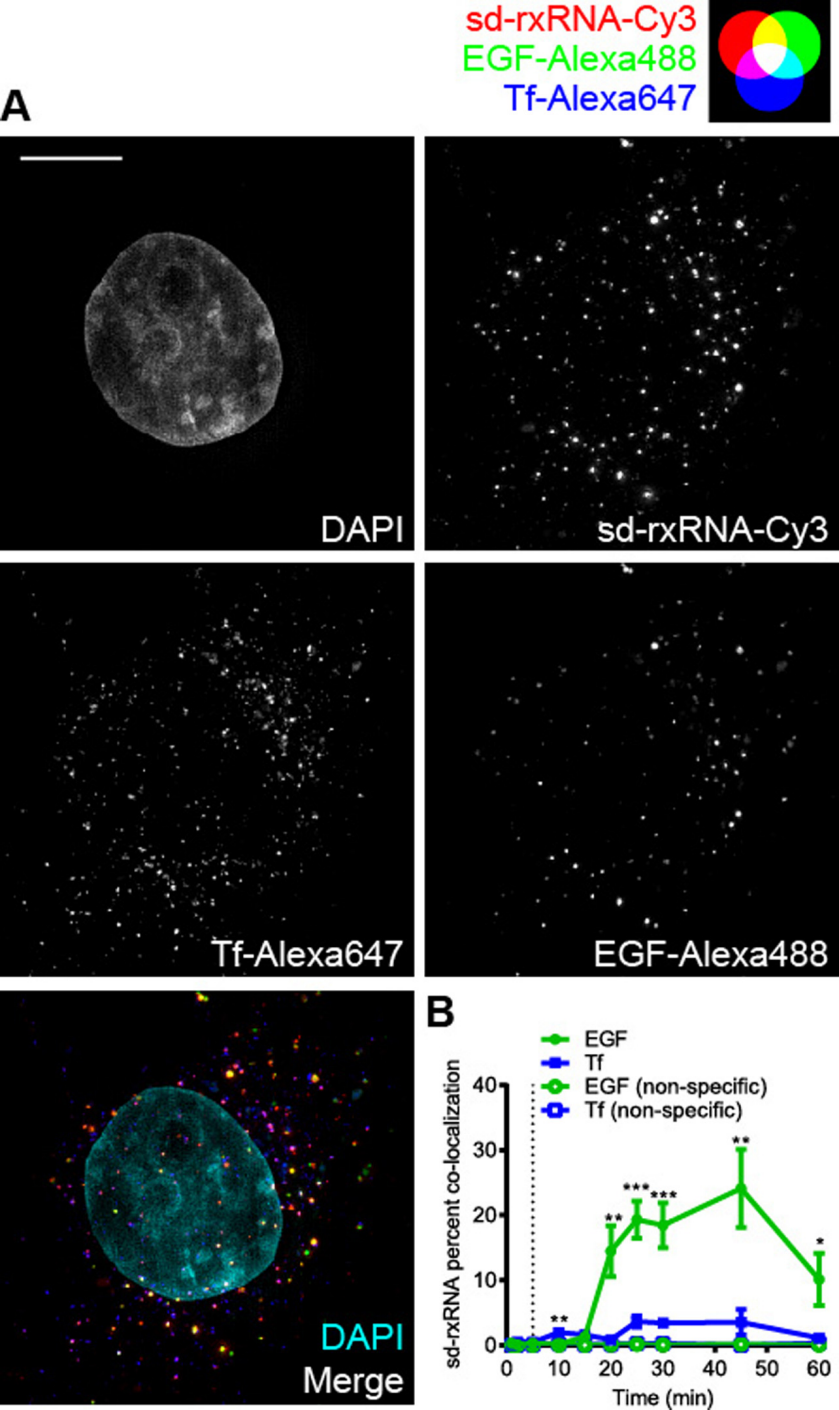
Quantitative analysis of sd-rxRNA co-trafficking with Tf and EGF shows preferential co-localization with EGF by fixed cell structured illumination microscopy. (**A**) COS-7 cells were grown on cover slips and simultaneously exposed to 25 ng/μl Tf-Alexa647, 250 nM sd-rxRNA-Cy3, and 1 ng/μl EGF-Alexa488 at 37°C and washed at 5 min. Cover slips were transferred to ice-cold PBS and then immediately fixed in 4% paraformaldehyde at the specified time points. Images were acquired by SIM and representative maximum intensity projections of cells fixed at 30 min are shown (see Supplementary Video 4). (**B**) Co-localization analysis of panel **A** is presented as mean ± SEM. Scale bar = 10 μm. Dotted line indicates wash with fresh media (*P < 0.05, **P < 0.01, ***P < 0.001, two-tailed Student's *t*-test).

In fixed cells, we again visually observed that sd-rxRNA preferentially co-localized with EGF compared to Tf (Figure 4A, yellow dots). Prior to 15 min, the majority of sd-rxRNA was associated with the plasma membrane outside the cell and the percent of sd-rxRNA co-localization with either EGF or Tf remained under 2%. However, by 20 min, the percent co-localization of sd-rxRNA with EGF increased to ∼14%. By 30 min, sd-rxRNA was clearly co-localizing preferentially with EGF compared to Tf as seen in a 3D projection of a cell (Supplementary Video 4). This trend continued and peaked at ∼24% by 45 min before falling back to ∼10% at 60 min. Conversely, the percent co-localization of sd-rxRNA with Tf increased to only ∼4% between 20 and 45 min. This significant difference persisted for all time points measured at 20 min and later (Figure 4B). Together, these results quantitatively confirm the initial observation from live cell imaging, suggesting that a major fraction of sd-rxRNA preferentially shares a trafficking pathway utilized for EGF internalization.

### sd-rxRNA preferentially associates with EEA1- but not RBSN-enriched endosomes

**Figure 5.**
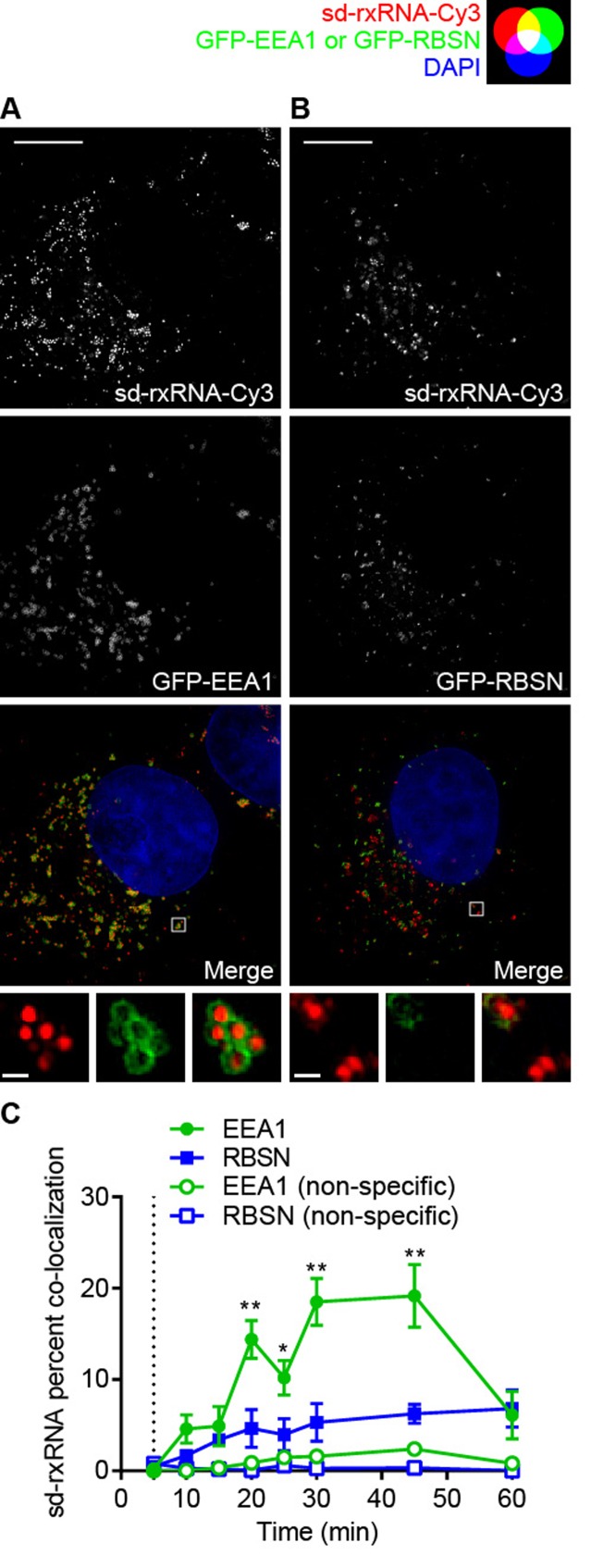
sd-rxRNA preferentially associates with EEA1- but not RBSN-enriched endosomes. COS-7 cells were grown on cover slips and transfected with either (**A**) GFP-EEA1 or (**B**) GFP-RBSN plasmids. All cells were grown on cover slips, kept at 37°C, treated with 250 nM sd-rxRNA-Cy3 and washed with fresh media at 5 min. At the specified time points, cover slips were transferred to ice-cold PBS and then immediately fixed in 4% paraformaldehyde. Images were acquired by SIM and representative maximum intensity projections of cells fixed at 30 min are shown. (**C**) Co-localization analysis of panels **A** and **B** are presented as mean ± SEM. Scale bar = 10 μm (inset scale bar = 500 nm). Dotted line indicates wash with fresh media (*P < 0.05, **P < 0.01, two-tailed Student's *t*-test).

In order to further dissect the sd-rxRNA trafficking pathway, we looked at two putative endosomal markers which have been implicated in EGF and Tf trafficking. Recent data suggests that EGF and Tf sorting occurs through endosomes enriched in early endosome antigen 1 (EEA1) and rabenosyn-5 (RBSN) (57), respectively. Consistently, EEA1 knockdown results in EGF missorting (37) and RBSN knockdown results in Tf missorting (39). To fluorescently tag these endosomal markers, COS-7 cells were transiently transfected with either GFP-EEA1 or GFP-RBSN fusion expressing plasmids (39). We first confirmed that there is little degree of co-localization between Tf and EGF (Supplementary Figure S4). Furthermore, EGF preferentially co-localizes with EEA1 and Tf preferentially co-localizes with RBSN (Supplementary Figure S5). Use of these cells expressing GFP fusions of endosomal markers allows evaluation of sd-rxRNA uptake in a non-perturbed state to ensure that observed preferential co-localization with EGF was not impacted by the initiation of EGF or Tf. The cells expressing GFP-EEA1 and GFP-RBSN (Figure 5) were treated with 250 nM sd-rxRNA-Cy3 and co-localization analysis was performed as described above (Figure 5A, 5B). sd-rxRNA showed a pattern of co-localization with EEA1-enriched endosomes similar to that of EGF, with levels steadily increasing to ∼15% after 15 min and peaking at ∼19% from 30 to 45 min before dropping to ∼6% at 60 min. Conversely, the percent co-localization of sd-rxRNA with GFP-RBSN was low (Figure 5C). Furthermore, when COS-7 cells were continuously incubated with sd-rxRNA for 22 hours, we again observed the same pattern of preferential co-localization with EEA1 compared to RBSN (Supplementary Figure S6). These results further support preferential trafficking of hydrophobic siRNAs through EGF/EEA1 involved pathways using an intracellular expressed marker system.

To test the functional relevance of these pathways, HeLa cells were first transfected with siRNAs targeting either EEA1, RBSN, or a non-targeting control (NTC) using RNAiMAX and then a dose response using sd-rxRNA targeting PPIB was performed. However, all transfected cells showed slightly enhanced sd-rxRNA silencing efficacy compared to untransfected cells (data not shown). Unfortunately, it is difficult to make a conclusion based on these results since any pre-treatment (transfection, electroporation, etc.) modifies or stresses the membrane composition, potentially altering the self-delivery pathway.

## DISCUSSION

Recently, significant effort in the siRNA field has focused on determining the mechanisms and pathways of oligonucleotide cellular uptake (58,59). It has been shown that when siRNA is delivered with LNPs in HeLa cells, only ∼100 loaded RISCs are required to achieve 50% gene silencing, which is significantly less than 1% of the total intracellular siRNA (11). This paradox of highly inefficient delivery to the site of action has raised the question of whether the precise mechanism by which RNAi-inducing compounds are delivered to cells plays a role in their silencing activity. Moreover, it is not clear whether all of the RNAi compounds taken up by cells can functionally engage with RISC, or whether distinct productive and non-productive uptake pathways exist. This information, and the elucidation of which pathways are productive, is required to systematically develop approaches to improve therapeutic potential. In addition, knowledge of intracellular pathways involved in silencing by RNAi oligonucleotides may inform on natural biological mechanisms of silencing.

Cholesterol conjugation has long been known to promote association with the cellular membrane, even with single-stranded oligonucleotides (60,61). Here, we study the internalization of sd-rxRNAs, hydrophobically modified siRNAs, which efficiently enter cells and tissues without requirement for the delivery vehicle. We use EGF and Tf as trafficking markers to get a better understanding of the intracellular fate of the oligonucleotide and mechanisms involved in the uptake. We observed preferential sd-rxRNA association with EGF trafficking which suggests the involvement of specific cell surface receptors. EGF internalization is dependent on rapid phosphorylation and ubiquitination of high affinity receptors. At low EGF concentrations, few receptors internalize, and their uptake takes place through clathrin-coated pits. However, at higher concentrations, most receptors internalize through macropinocytosis (62,63).

Hydrophobic siRNA internalization is very quick and saturable. Pre-incubation of cells just for ten minutes with non-labeled compound of identical chemical composition chased with the labeled oligonucleotides effectively blocks labeled compound internalization without any significant effect on membrane binding. This supports a two-step mechanism with membrane binding and cellular uptake being functionally independent events. Membrane binding is likely driven by overall hydrophobicity of the oligonucleotide and cholesterol ability to intercalate into the membranes, consistent with initial diffuse, non-saturable and reversible membrane staining. Then, the compounds are internalized through a hypothetical receptor interaction and significantly overlap with EGF-containing endosomes. The second step is fully saturable within minutes. Three to six hours is required for the machinery involved in internalization to be recycled or resynthesized. This observation could potentially inform on a better oligonucleotide dosing regimen *in vivo*.

The study of the cellular uptake mechanisms of oligonucleotides is complicated by their requirement for endocytosis to enter the cell and need to exit from these organelles to gain further access into the cytoplasmic space. The endocytic pathway in mammalian cells is highly complex; cargo may be internalized from the plasma membrane by several different mechanisms, and following internalization enter a system of endosomes, which are numerous, dynamic and highly heterogeneous in size, shape, composition and location (35,64–70). Thus, defining the pathways of oligonucleotide entry requires techniques that can monitor the compound of interest relative to the plasma membrane and distinct endosome classes with sufficient temporal and spatial resolution. TIRF microscopy provides a high resolution, sensitive method to monitor the localization of fluorophores in the vicinity of the plasma membrane, where all initial events of endocytosis take place. The spatial resolution of TIRF is further enhanced by information regarding the exact brightness of visible objects obtained by SIM. Using these optical approaches, visualization of two extensively studied systems—Tf and its receptor (TfR), and EGF and its receptor (EGFR) (40,71–73)—has revealed that these ligands follow distinct trafficking pathways immediately after internalization (37,38).

The mechanisms by which internalized sd-rxRNA achieves target silencing must involve its interaction with cytoplasmic RISC. Previous reports suggest that components of RISC are associated with multi-vesicular body membranes, and that depletion of multi-vesicular bodies impairs silencing activity (74,75). EEA1 is associated with endosomes that contain cargo destined for lysosomes, which in turn involves the formation of multi-vesicular bodies (76). This interpretation is supported by studies in which depletion of hepatocyte growth factor-regulated tyrosine kinase substrate (Hrs) (77), which is involved in multi-vesicular body formation, results in both impaired EGFR degradation (78) and diminished miRNA silencing (75). Conversely, knocking down members of the ESCRT-I complex has also been shown to increase both *in vitro* and *in vivo* efficacy of anti-miR compounds (miRNA inhibitors), although its role in the context of antisense oligonucleotide or siRNA delivery is still unclear and warrants further study.

Once internalized, EGF receptors are associated with endosomes characterized by the presence of EEA1 (37,41–43,79–80). The co-localization of sd-rxRNA with EGF is consistent with direct RNA-receptor interactions. RNA binding to receptors has been reported, for example, double stranded RNA 20-30-mers bind to pattern recognition receptors (81,82). Whether these receptors are activated by sd-rxRNA, how they are internalized and the degree of overlap with cholesterol trafficking is a subject of further evaluation. The preferential internalization of oligonucleotides into specific endosomes may be relevant for its biological activity, as different endosome classes are associated with different fates; Tf internalization, which involves endosomes characterized by the presence of RBSN (39,83), is followed by recycling to the plasma membrane, while the internalization of EGF into endosomes containing EEA1 is followed by lysosomal degradation (37,42,80). In fact, it has been shown that ∼70% of internalized siRNAs are effluxed out of the cell by a Niemann-Pick type 1 (NPC1) pathway, suggesting that efficacy could be improved by escaping these recycling pathways (26). Furthermore, another recent paper suggested that endosomal escape occurs at a very low frequency and only from a specific subset of endosomes (13). However, both of these studies used LNP-mediated siRNA delivery, so how relevant these observations may be for self-delivering hydrophobically modified siRNAs remain to be seen.

**Figure 6.**
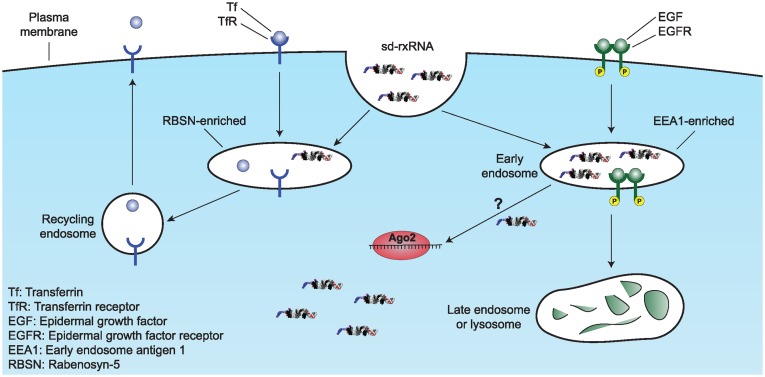
Proposed model of sd-rxRNA internalization and trafficking. EGF traffics through EEA1-enriched endosomes and is sent to the lysosome for degradation while Tf traffics through RBSN-enriched endosomes and is recycled back to the plasma membrane. A fraction of internalized sd-rxRNA preferentially traffics through EEA1-enriched endosomes, similar to EGF. In order to achieve target silencing, sd-rxRNA must undergo endosomal escape in order to reach the cytoplasm where the components of RISC, such as Argonaute 2 (Ago2), are located; however, this mechanism is still not known.

In summary, we show that the internalization of sd-rxRNAs, a class of hydrophobically modified siRNAs, is a quick, two-step process, involving membrane binding and saturable preferential sorting into an EGF-EEA1-like pathway (Figure 6). However, it is important to note that accumulation of sd-rxRNA in EEA1-containing endosomes does not imply that this is a productive pathway and thus a preferable uptake mechanism for their silencing activity. Rather, this is a method to visualize the internalization and trafficking of these molecules and can be used to further investigate other conjugates and pathways.
